# Specific tests for nasal permeability

**DOI:** 10.1016/S1808-8694(15)31114-9

**Published:** 2015-10-20

**Authors:** Renato Roithmann

**Affiliations:** Brazilian Lutheran University

The definition of a normal nose, or rather what it means to breathe normally through the nose, is very controversial in the literature. Some schools use purely subjective criteria in order to evaluate their patients and/or volunteers in research projects. In such situations, it is the patient/volunteer who defines if he/she breathes well or not through both or only one nostril. Other schools try to quantify this subjective nasal breathing by applying scales. One example is the visual-analogue scale, in which the patient/volunteer marks an x on a 0 to 100 mm line as to how one sees one's breathing. Answers vary from “my nose is fully opened to my nose is fully obstructed. A numeric value is then generated and compared before and after the most varied interventions (e.g. nasal surgeries or medication tests).

Therefore, to conclude on the nasal respiratory function in a given clinical or research situation based only on the individual's perception in the test does not seem to suffice. Factors such as the individual's emotional status, among others, may influence breathing perception and induce imprecise responses on the real breathing function of one's nose.

Thus, usually, besides the individual's own perception, rhinoscopy is an essential exam in order to determine nasal permeability.

So far, so good. Now, how can one know if a septum deviation or congested nasal conchae seen during rhinoscopy would have a greater or lesser impact on nasal breathing? The search for this answer has troubled many generations of researchers so far.

Increasingly more sophisticated image exams are able to minutely define the nose's anatomical structure and neighboring areas at the time of investigation. Nonetheless, the isolate observation of a CT scan does not provide the examiner a proper definition of a normal or abnormal nose as far as breathing is concerned. In other words, both rhinoscopy and image exams do not quantify, and alone are not able to differentiate, a normal from an abnormal nose, from the respiratory standpoint.

Specific tests for nasal permeability have been used for decades now in order to try and quantify the ever so complex symptom of nasal obstruction. The Glatzel and Gertner mirrors, expiratory and inspiratory flow measuring devices modified for nasal exams, oscillometry, and other tests try to reach this answer. Nonetheless, it was the rhinomanometry, and most recently the acoustic rhinometry; the most used tests in research centers in this field, and are today considered specific tests for nasal permeability. Rhinomanometry is a dynamic exam that quantifies transnasal air flow and provides the known index of nasal resistance. Acoustic rhinometry is a static test that quantifies the nasal cross section area that is used to calculate nasal volume. These two tests then provide distinct parameters of nasal permeability and complete one another. Both quantify the magnitude of nasal obstruction at a given time. Notwithstanding, these tests do not provide the etiological diagnosis in nasal obstructions. Moreover, the isolate interpretation of the values encountered is unable to differentiate the normal from the abnormal nose because of the dynamic behavior of the nasosinusal mucosa (physiological nasal cycle, etc…).

New studies, as the one presented in this issue of our journal, try to define values in normal populations, and are most relevant for the development and better understanding of the results attained.

Thus, what is the state of art in terms of nasal breathing evaluation? The safest ground for both the clinician and the researcher in their daily routines seems to lie in checking all the available factors, that is, consider clinical history (breath well or not), rhinoscopy and/or image studies, and the results from the specific tests of nasal permeability (rhinomanometry and/or acoustic rhinometry).

